# Dissecting Nucleosome Function with a Comprehensive Histone H2A and H2B Mutant Library

**DOI:** 10.1534/g3.117.300252

**Published:** 2017-10-16

**Authors:** Shuangying Jiang, Yan Liu, Caiyue Xu, Yun Wang, Jianhui Gong, Yue Shen, Qingyu Wu, Jef D. Boeke, Junbiao Dai

**Affiliations:** * Ministry of Education Key Laboratory of Bioinformatics, School of Life Sciences, Tsinghua University, Beijing 100084, PR China; † Center for Synthetic and Systems Biology, School of Life Sciences, Tsinghua University, Beijing 100084, PR China; ‡ Center for Synthetic Biology Engineering Research, Shenzhen Institutes of Advanced Technology, Chinese Academy of Sciences, Shenzhen 518055, China; § China National GeneBank, Beijing Genomics Institute-Shenzhen, Shenzhen 518120, China; ** Beijing Genomics Institute-Shenzhen, Shenzhen 518083, China; †† Institute for Systems Genetics, New York University Langone Medical Center, New York 10011; ‡‡ Department of Biochemistry and Molecular Pharmacology, New York University Langone Medical Center, New York 10011

**Keywords:** histone, heterochromatin gene silencing, DNA damage, post-translational modification

## Abstract

Using a comprehensive library of histone H2A and H2B mutants, we assessed the biological function of each amino acid residue involved in various stress conditions including exposure to different DNA damage-inducing reagents, different growth temperatures, and other chemicals. H2B N- and H2A C-termini were critical for maintaining nucleosome function and mutations in these regions led to pleiotropic phenotypes. Additionally, two screens were performed using this library, monitoring heterochromatin gene silencing and genome stability, to identify residues that could compromise normal function when mutated. Many distinctive regions within the nucleosome were revealed. Furthermore, we used the barcode sequencing (bar-seq) method to profile the mutant composition of many libraries in one high-throughput sequencing experiment, greatly reducing the labor and increasing the capacity. This study not only demonstrates the applications of the versatile histone library, but also reveals many previously unknown functions of histone H2A and H2B.

In eukaryotes, genomic DNA is compacted into chromatin to fit into the nucleus. As the functional unit of chromatin, a nucleosome consists of histone octamers—including two copies each of the four core histones H2A, H2B, H3, and H4—and ∼147 bp DNA (Luger *et al.* 1997). Histones are known to be modified at multiple positions and numerous studies have been carried out in recent years to understand the function and regulation of these post-translational modifications (PTMs) (Morrison *et al.* 2004; Keogh *et al.* 2005; Masumoto *et al.* 2005; Shilatifard 2006; Kouzarides 2007; Chang and Pillus 2009; Dang *et al.* 2009; Mosammaparast and Shi 2010; Zhou *et al.* 2011; Rando 2012; Wurtele *et al.* 2012; Patel and Wang 2013; Watanabe *et al.* 2013; Zentner and Henikoff 2013; Tessarz and Kouzarides 2014; Sen *et al.* 2015). To date, ∼20 types of PTMs, which are distributed among >100 residues of core histones, have been identified (Tan *et al.* 2011; Huang *et al.* 2014). Although several “popular” modifications have been well studied, the functions of many newly discovered histone marks remain poorly understood. In addition, critical functions of residues without any PTMs have also been reported (Luo *et al.* 2010; Yu *et al.* 2011), underscoring the importance of each histone residue.

Compared to histone H3 and H4, histone H2A and H2B are less conserved from yeast to human, especially in the N-terminal tails. In addition, H3–H4 tetramers form the core of the nucleosomes while H2A and H2B are more readily displaced (Kimura and Cook 2001; Kireeva *et al.* 2002). Perhaps for these reasons, fewer studies have focused on histones H2A and H2B than for H3 and H4. Although modifications at >50 residues of H2A and H2B have been characterized (Tan *et al.* 2011; Huang *et al.* 2014), only a few modified residues have been well characterized. For example, ubiquitination of histone H2B K123, mediated by Rad6, stimulates the methylation of H3K4 and H3K79 and plays important roles in transcription regulation and DNA damage checkpoint signaling (Robzyk *et al.* 2000; Ng *et al.* 2002; Sun and Allis 2002; Giannattasio *et al.* 2005; Pavri *et al.* 2006; Fleming *et al.* 2008; Moyal *et al.* 2011; Hung *et al.* 2017). The functions and regulation of most modifications remain unknown. Until now, only two versions of the histone H2A and H2B mutant library have been available for *Saccharomyces cerevisiae* (Matsubara *et al.* 2007; Nakanishi *et al.* 2008; Sakamoto *et al.* 2009), and both of these libraries consist of exclusively alanine substitutions and can only be used in an episomal plasmid format.

In this study, we used a comprehensive new library of histone H2A/H2B mutants to systematically probe the functions of histone H2A/H2B residues in different biological processes, including DNA damage repair, temperature tolerance, genome stability, and heterochromatic gene silencing, revealing many interesting features of these less well-known histones. In addition, the presence of two unique barcodes on every mutant allowed the adoption of a bar-seq method to dissect complex phenotypic assays, which otherwise would be labor intensive and time-consuming. Other applications, such as construction of an interaction network among the core histone surfaces, can be envisioned.

## Materials and Methods

### Yeast strains, plasmids, and media

Strains in the BY-H2ML1&2 library were used for high-throughput phenotype analysis under different stress conditions with serial dilution (SD). Linearized plasmids of H2ML1 by *Bci*VI were transformed into JDY187 (a derivative of strain GRF167) and SHY15 (a derivative of W303) to screen the mutants that affect heterochromatin silencing and genome stability, respectively. Strains in the BY-H2ML1 library were used for methyl methanesulfonate (MMS) assay by bar-seq or SD. Genotype of all strains are listed in Supplemental Material, Table S1 in File S1.

Synthetic complete (SC) medium contained 0.17% (w/v) yeast nitrogen base without amino acids and ammonium sulfate (YNB, USBio), 0.5% (w/v) ammonium sulfate, 2% (w/v) glucose, and 2% (w/v) bacto-agar, supplemented with 0.2% (w/v) amino acids drop-out powder. The concentration of 5-FOA in synthetic media was 1 g/L. Drug-containing media were prepared by supplementing YPD with 200 mM hydroxyurea (HU), 0.03% (v/v) MMS, 10 μg/ml benomyl, 8 μg/ml camptothecin (CPT), 2 μg/ml nocodazole, 25 nM rapamycin, or 0.3% (v/v) acetic acid, respectively.

### Chromosome loss assay

The reporter strain (SHY015) was derived from SBY8054, which contains an artificial chromosome III fragment with *SUP11* and *HIS3* markers (Ng *et al.* 2013). The *HTA2-HTB2* locus was knocked out. *ura3-1* and *leu2-3* were corrected to *ura3*∆*0* and *leu2*∆*0* to generate the final strain. Individual histone mutations were integrated at the endogenous *HTA1-HTB1* locus and confirmed by colony PCR as described above. The chromosome loss assay was performed as previously described (Ng *et al.* 2013). At least two independent isolates were tested for each mutant.

### Heterochromatin silencing assay

The reporter strain (JDY187) was derived from YNB12, containing two makers, *MET15* and *ADE2*, within the rDNA repeat and the subtelomeric region on chromosome V, respectively (Smith *et al.* 1999). Genomic *HTA1-HTB1* and *HTA2-HTB2* were knocked out and the cells were supplied with pJD78 to support viability, which was removed after a histone mutant was introduced. At least two independent isolates were tested for each mutant. For rDNA silencing, diluted fresh cells were plated on lead plates (1 g/L) for 1 wk at 30° before we scored the colony color (Dai *et al.* 2010). For telomeric silencing, cells were spotted on SC–Leu plates and incubated at 30° for 3 d. Pictures of the red colony color were taken after storing the cells at 4° for 4 d.

### Identification of MMS-sensitive alleles with bar-seq

Viable H2A/H2B mutants were pooled together and stored in 15% glycerol at −80°. For the MMS assay, cells of the H2A/H2B pool were inoculated in 50 ml of YPD for 4 hr at 30°. Next, 10 ml of cell culture was transferred into two new tubes: one with MMS (final concentration at 0.05%) and the other without. The cells were cultured at 30° for 2 hr, precipitated by centrifugation, and washed with ddH_2_O twice before inoculation into 50 ml YPD for overnight growth at 30°. The cell density of the overnight cultures was measured and 2.5 × 10^8^ cells from both cultures were harvested for genomic DNA preparation.

For bar-seq, genomic DNA was isolated and used as the template for two rounds of PCR to construct the sequencing libraries. At first, the TAG regions of each histone mutant were amplified using primers with same the annealing sequences (Table S6 in File S1) (Dai *et al.* 2008). To sequence different samples, a five-digit index sequence, among which at least two nucleotides were different from each other, were introduced. Next, the Illumina sequencing adaptors (P5/P7) were added by a second round of PCR using diluted first PCR products as templates. These PCR products were mixed, gel purified, and subjected to Illumina GAII pair-end sequencing. We got over 95k pairs of reads per sample. Statistics of log2 and *P*-value with FDR/FWER false positive control were used to compare the sequencing data for different mutants in MMS-treated and YPD group. The mutants with log2 ratio < −1, *P* < 0.01, *q* < 0.01 were defined as MMS-sensitive mutants.

### Data availability

The yeast strains are available upon request and will be deposited with ATCC. Plasmids will be deposited with Addgene. File S1 contains detailed descriptions of all supplemental data, including five supplemental figures and six supplemental tables. Table S3 contains the complete list of results from testing all strains in the BY-H2ML1&2 library with various assays.

## Results

### High-throughput phenotype analysis of a yeast library containing two copies of integrated histone genes

After constructing the systematic yeast strain library in which each cell contains two copies of the same mutant histone, under the control of their native promoters (*HTA1-HTB1* and *HTA2-HTB2* promoter) and integrated at the endogenous loci, respectively, a series of phenotypic analyses was performed. Four classes of growth conditions were evaluated, including: (i) stresses that lead to DNA damage (HU, MMS, or UV irradiation), (ii) microtubule destabilization (benomyl and nocodazole), (iii) temperature stresses (16 and 39°), and (iv) others (rapamycin and acetic acid) (Table S2 in File S1). The degree of sensitivity of H2A/H2B mutants under each condition was arbitrarily classified (from 1 to 4), representing increased sensitivity. The complete phenotype data are listed in Table S3.

**Figure 1:**
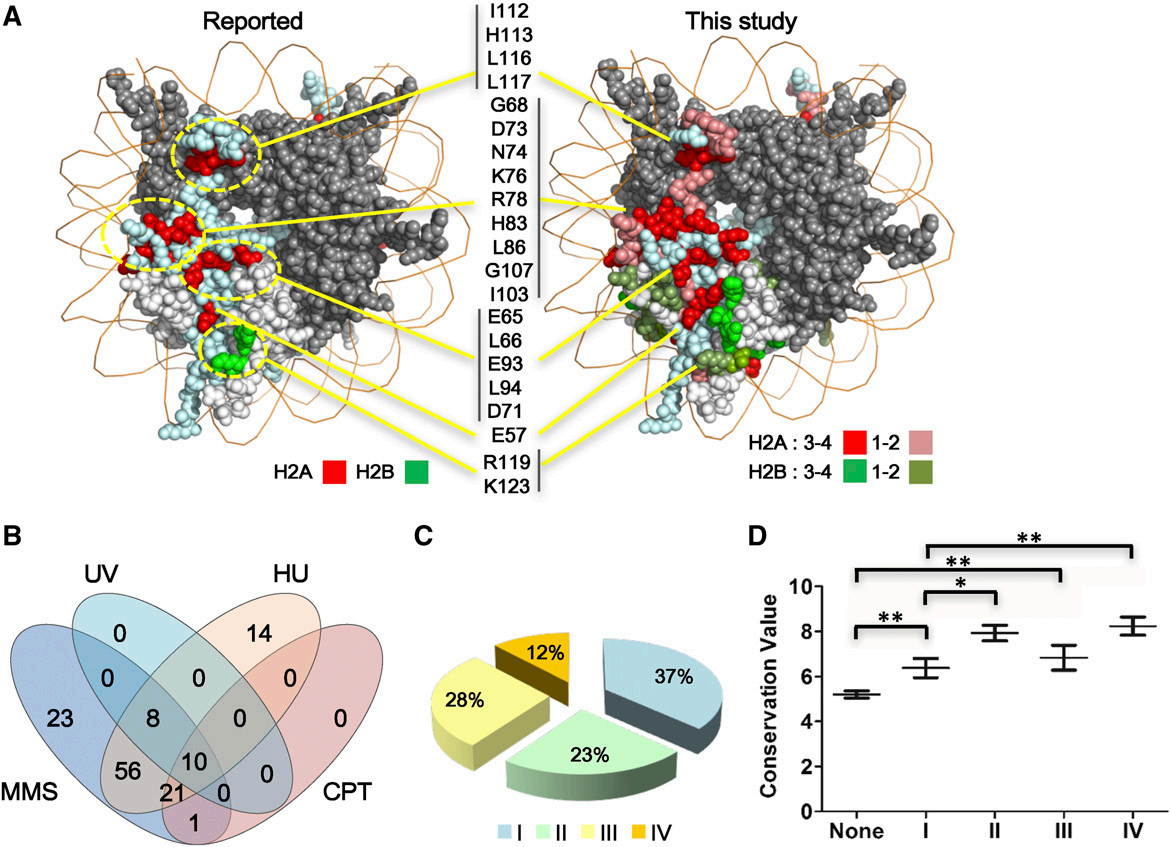
High-throughput phenotype analysis of a yeast library containing two copies of histone mutants. (A) More HU-sensitive mutants were identified with the BY-H2ML1&2 library. The reported mutants (Matsubara *et al.* 2007; Sakamoto *et al.* 2009) were marked in bright red for H2A and bright green for H2B. For BY-H2ML1&2, H2A/H2B mutants were marked with different red/green colors, indicating the strength of phenotype, which was defined by using a number from 1 to 4 (to indicate weak to strong phenotype). Bright red: H2A mutants with phenotypes classified 3 or 4. Russet-red: H2A mutants with phenotypes classified 1 or 2. Bright green: H2B mutants with phenotypes classified 3 or 4. Dull green: H2B mutants with phenotypes classified 1 or 2. If two or more mutants for a certain residue showed phenotypes, the highest phenotype degree of this residue was displayed. H2A R30 is not shown since it cannot be visualized at this angle. The *S. cerevisiae* nucleosome structure is used in the entire paper (PDB number: 1ID3). The locations of corresponding clusters of mutants in the nucleosome structure were marked with yellow lines and circles. (B) Venn diagram of mutants sensitive to different DNA damage-inducing stresses. (C) Statistical analysis of mutants with phenotypes according to their biological processes. See the classification of different stresses in Table S2 in File S1. I: mutants with one class of phenotype, blue; II: mutants with two classes of phenotypes, light green; III: mutants with three classes of phenotypes, yellow; and IV: mutants with four classes of phenotypes, orange. (D) Evolutionary conservation scores for mutants with different classes of phenotypes were calculated using ConSurf. The definition of phenotype numbers I–IV is the same as those in (C). Data were represented as mean ± SEM. * *P* < 0.05 and ** *P* < 0.01. CPT, camptothecin; MMS, methyl methanesulfonate; HU, hydroxyurea; UV, ultraviolet.

In total, about a quarter of the mutants showed at least one phenotype (150/570), suggesting that histone H2A and H2B are important in maintaining normal nucleosome function. The positions of the mutated residues with phenotypes were mapped to the nucleosome structure and represented by different colors (Figure 1A and Figure S1B in File S1). For example, we identified 109 mutants covering 76 amino acid residues that showed sensitivity to HU to different extents. When compared to previous screens (Matsubara *et al.* 2007; Sakamoto *et al.* 2009), we not only identified all the reported residues, but also discovered many other critical ones (Figure 1A). Similar results were obtained for the mutants sensitive to MMS (Figure S1A in File S1). Additionally, it is notable that two mutants of native alanine residues, H2A A61S and A70S (a residue commonly omitted from most mutagenesis studies), were found to be sensitive not only to DNA damage reagents, but also to drugs that disrupt microtubules (Table S3). Furthermore, besides testing if the yeast strains could survive at higher temperature (39°), we screened the mutants that were sensitive to lower temperature (16°). These mutants could potentially be applied to study the mechanisms of adaptation to low temperature, which remain largely unknown (Aguilera *et al.* 2007). Together, these results indicated that the new histone H2A and H2B library, with a greatly expanded mutant and phenotype spectrum, could serve as a valuable resource to reveal a functional landscape with increased resolution for the two core histones.

Interestingly, among the four different DNA damage stresses, MMS, UV, HU, and CPT, we found that mutants sensitive to UV and CPT were subsets of those sensitive to MMS stress. Meanwhile, several mutants showed sensitivity specifically to MMS or HU (Figure 1B). It is notable that several mutants of C-terminal deletion of H2A were specifically sensitive to MMS (Table S4 in File S1). Furthermore, among the mutants with phenotypes, >60% of mutants showed at least two classes of phenotype (Figure 1C), which suggests that these residues are involved in at least two important biological processes. The conservation scores of each mutant with different classes of phenotypes were analyzed. From Figure 1D, it is obvious that the residues with two or more classes of phenotypes are more conserved than those that showed only one or none.

**Figure 2:**
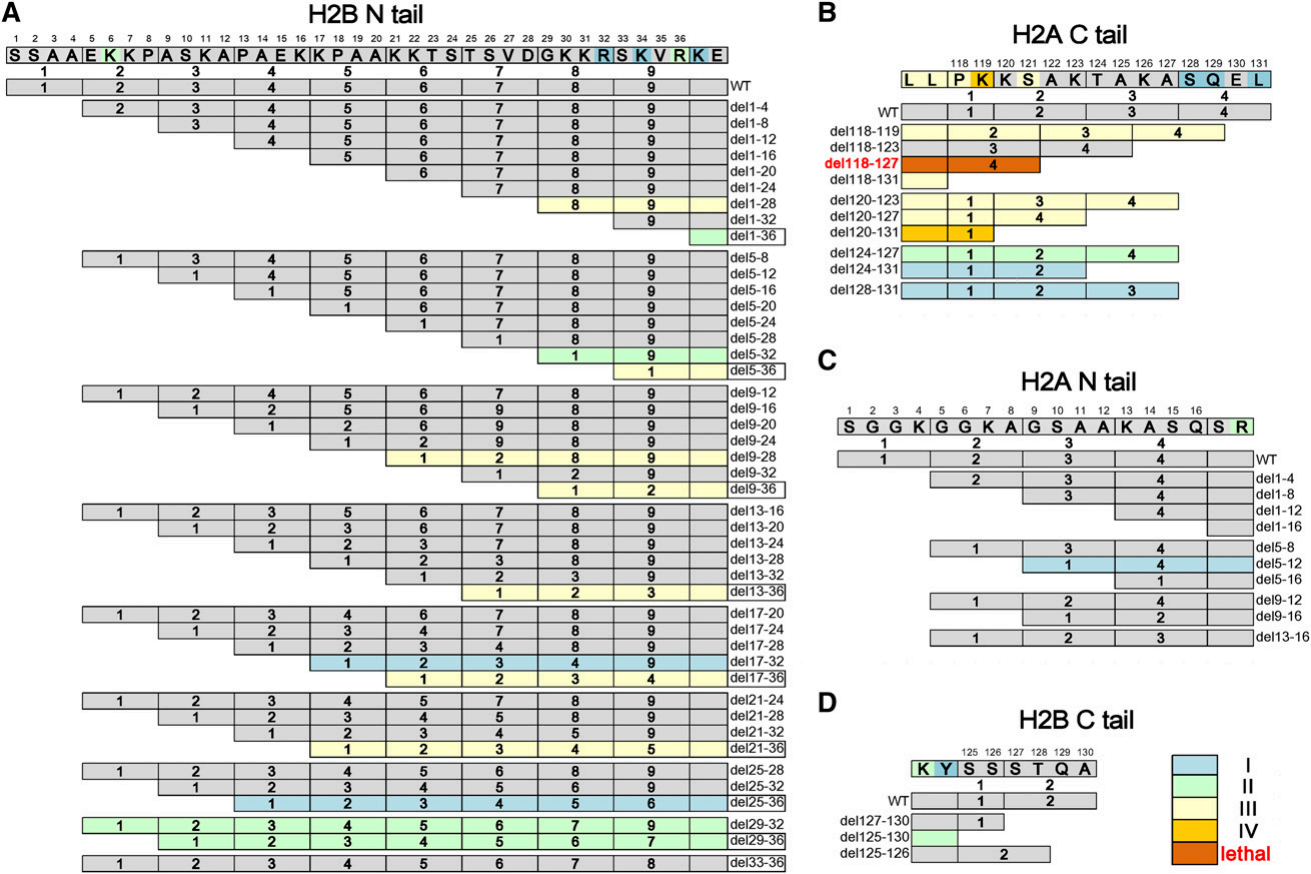
Phenotype analysis for histone tails of H2A and H2B. (A) The phenotypes of deletion mutants of H2B N terminal tail; (B) The phenotypes of deletion mutants of H2A C terminal tail; (C) The phenotypes of deletion mutants of H2A N terminal tail; (D) The phenotypes of deletion mutants of H2B C terminal tail. For simple visualization, the amino acids within histone tails were divided into different units based on the mutagenesis strategy. The numbers of phenotypes for these mutants are marked with the same colors used in Figure 1C. I: mutants with one class of phenotype, blue; II: mutants with two classes of phenotypes, light green; III: mutants with three classes of phenotypes, yellow; and IV: mutants with four classes of phenotypes, orange. The lethal mutant H2A del118-127 is marked with a brown color and red characters. Mutants without any phenotypes are shown in gray. Point mutants with phenotypes within these histone tails are also labeled.

When we summarized the phenotypes of histone tail deletions, single mutants with different phenotypic classes were also colored (Figure 2). It is remarkable to find that deletions of the histone H2B N- and H2A C-termini showed many phenotypes, including lethality (Figure 2, A and B), whereas almost none of the H2A N- and H2B C-terminal mutants had any defects (Figure 2, C and D), suggesting that these tails were functionally distinct, at least in the processes tested. This is different from the case of the H3 and H4 N-terminal tails, which actually show functional redundancy in many assays. These data are consistent with the fact that there were many fewer point mutants with phenotypes in the H2A N- and H2B C-terminal tails than in the other tails (Figure 2, C and D). In contrast, among the H2B N-terminal tail mutants, eight out of nine deletions from which residues 33–36 were removed (marked with squares, Figure 2A) showed phenotypes, implying that this region is critical for nucleosome function. Paradoxically, when only those four residues (del33-36) were deleted, the cells were phenotypically normal. Upon sequence analysis, we observed that amino acids 29–32 are similar to 33–36 within the tail and both conform to a “XKXR” motif, where X represents residues that are not negatively-charged (Figure S2A in File S1). Deletion of residue 33–36 moves amino acids 29–32 to the same position, where they might fulfill a similar function. From the nucleosome structure, R36, a conserved residue from yeast to human, interacts with the minor groove of surrounding DNA and the H2B N-terminal tail protrudes out of the nucleosome between two strands of DNA (Figure S2 in File S1). The positively-charged K and R within the XKXR motif at this location might mediate interactions between histone H2B and DNA, thereby stabilizing the nucleosome structure. In support of this hypothesis, point mutations at either residue (K34E of R36E) also showed DNA-related phenotypes (Table S3). Since these residues were previously mutated only to alanine, none of these residues were identified in previous studies (Matsubara *et al.* 2007; Sakamoto *et al.* 2009). Therefore, we conclude that it is essential to remove both copies of this motif to get the phenotype correlated with nucleosome instability.

### Histone H2A and H2B mutants affecting genome stability

**Figure 3:**
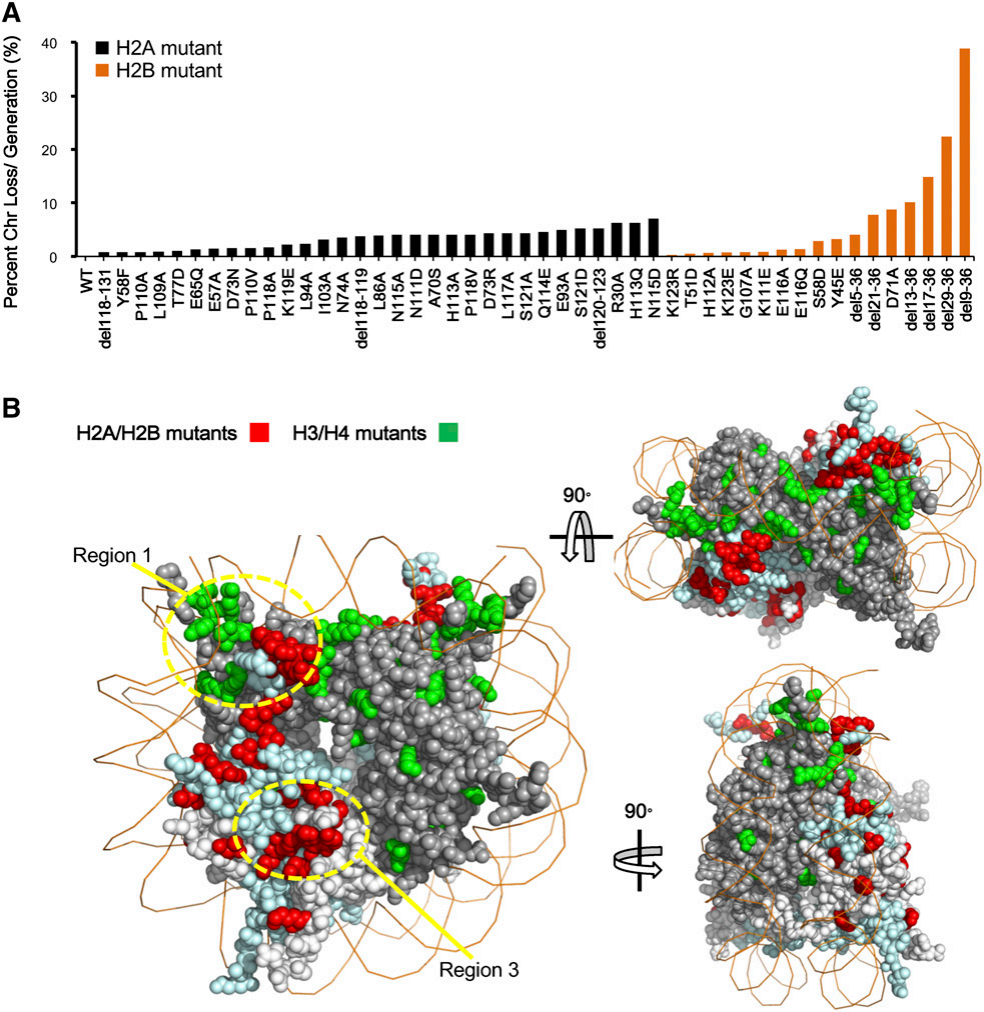
Identification of histone H2A and H2B mutants affecting genome stability. (A) The frequency of chromosome (Chr) loss for the indicated histone H2A mutants (black) and H2B mutants (orange). (B) The distribution of mutants with elevated frequency of chromosome loss in four core histones. Mutants in histone tails and the compound substitutions are not shown. The picture in the upper right side is the top view of the nucleosome and the lower one is the lateral view. H2A/H2B mutants are marked in bright red and H3/H4 mutants are marked in bright green.

Our previous study indicated that many residues on histone H3 and H4 are required to maintain a stable genome (Ng *et al.* 2013). However, the contribution of histones H2A and H2B to genome stability has not been explored. Therefore, the same chromosome loss assay was employed. We defined a mutant to be significant if, compared to wild-type, more than a fourfold of increase of chromosome loss rate per generation was found. Among the 592 mutants, a total of 31 alleles in H2A and 17 alleles in histone H2B were identified that met or exceeded this arbitrary threshold (Figure 3A). The affected residues were mapped onto the nucleosome structure. Besides histone H2A and H2B, we also highlighted the histone H3 and H4 residues from the previous study (Ng *et al.* 2013) and newly identified ones (Figure S3A in File S1), giving a much more comprehensive view of residues involved in genome stability in all four core histones (Figure 3B).

Several nucleosome regions were revealed by this analysis. (1) Residues near the DNA entry and exit sites, such as mutations in the H3 α N helix and the nearby H2A C-terminal (Figure 3, A and B), are important for genome stability. This region is known to be required for Sgo1p, a key modulator of chromosome biorientation, to dock onto the nucleosome (Luo *et al.* 2010; Kawashima *et al.* 2011). The mutants may attenuate Sgo1p binding, subsequently leading to defects in chromosome segregation. (2) The N-terminal tails of two histones, H2B and H4, are particularly important for maintaining a stable genome. We found 6 and 11 deletion mutants on the N-termini of histone H2B and H4, respectively, that led to severe chromosome loss (Figure 3A and Figure S3A in File S1) (Ng *et al.* 2013). We analyzed the distribution of chromosome loss rates for all core histones and found that the mean loss rates of histone H4 and H2B were higher than those of H3 and H2A (Figure S3B in File S1), which were mainly contributed from the N-terminal deletion mutants of these two histones (Figure 3A and Figure S3A in File S1) (Ng *et al.* 2013). (3) Mutants of the acidic patch on histone H2A, which interacts with the N-terminal tail of histone H4, showed an increased frequency of chromosome loss. We hypothesize that the above two regions might be correlated since a likely charge-dependent interaction between H2A and the H4 tail is important for the formation of 30 nM chromatin fibers (Luger *et al.* 1997; Dorigo *et al.* 2004; Song *et al.* 2014). To test this hypothesis, we replaced the first 24 amino acids of histone H4 with that of histone H3, which has a different amino acid sequence but a similar number of positively-charged residues (Figure S4A in File S1). As expected, the chimeric H4 significantly rescued the chromosome loss phenotype. We further showed that the positively-charged lysine residues in the interaction region are required since mutations of these residues led to a failure to rescue the phenotype (Figure S4B in File S1). (4) Finally, there are a few mutants, such as H3 K115A and H2A T77D, which locate approximately to the DNA–histone interface, which were also identified. These mutants might the loosen interaction between histones and DNA, leading to unstable nucleosomes.

### Histone mutants that affect heterochromatin silencing

Although the three heterochromatin regions all show repressive transcription of reporter genes, silencing at rDNA, silent mating loci, or subtelomeric regions requires molecularly distinct complexes (Huang and Moazed 2003; Grunstein and Gasser 2013), indicating the existence of apparent distinct mechanisms in these regions. Many residues on histones H3 and H4 are known to be involved in regulating heterochromatic gene silencing (Kimura *et al.* 2002; Park *et al.* 2002; Richards and Elgin 2002; Suka *et al.* 2002; Sun and Allis 2002; Ng *et al.* 2003; Dai *et al.* 2008; Norris *et al.* 2008; Norris and Boeke 2010), whereas the function of histone H2A and H2B on heterochromatic gene silencing remains poorly studied. Here, to dissect the function of H2A and H2B in the silencing of the subtelomere and rDNA region, a strain containing subtelomeric *ADE2* and *MET15* within the rDNA repeat was used as the reporter strain (Smith *et al.* 1999). We systematically tested the expression of the two normally repressed reporters in each histone H2A and H2B mutant. Table S5 in File S1 shows the full list of mutants identified in this screen, including many previously reported residues, such as H2B K123, T122, K111, R119, R102, and R95 (Ng *et al.* 2002; Sun and Allis 2002; Chandrasekharan *et al.* 2010; Dai *et al.* 2010; Kyriss *et al.* 2010; Wang *et al.* 2011).

**Figure 4:**
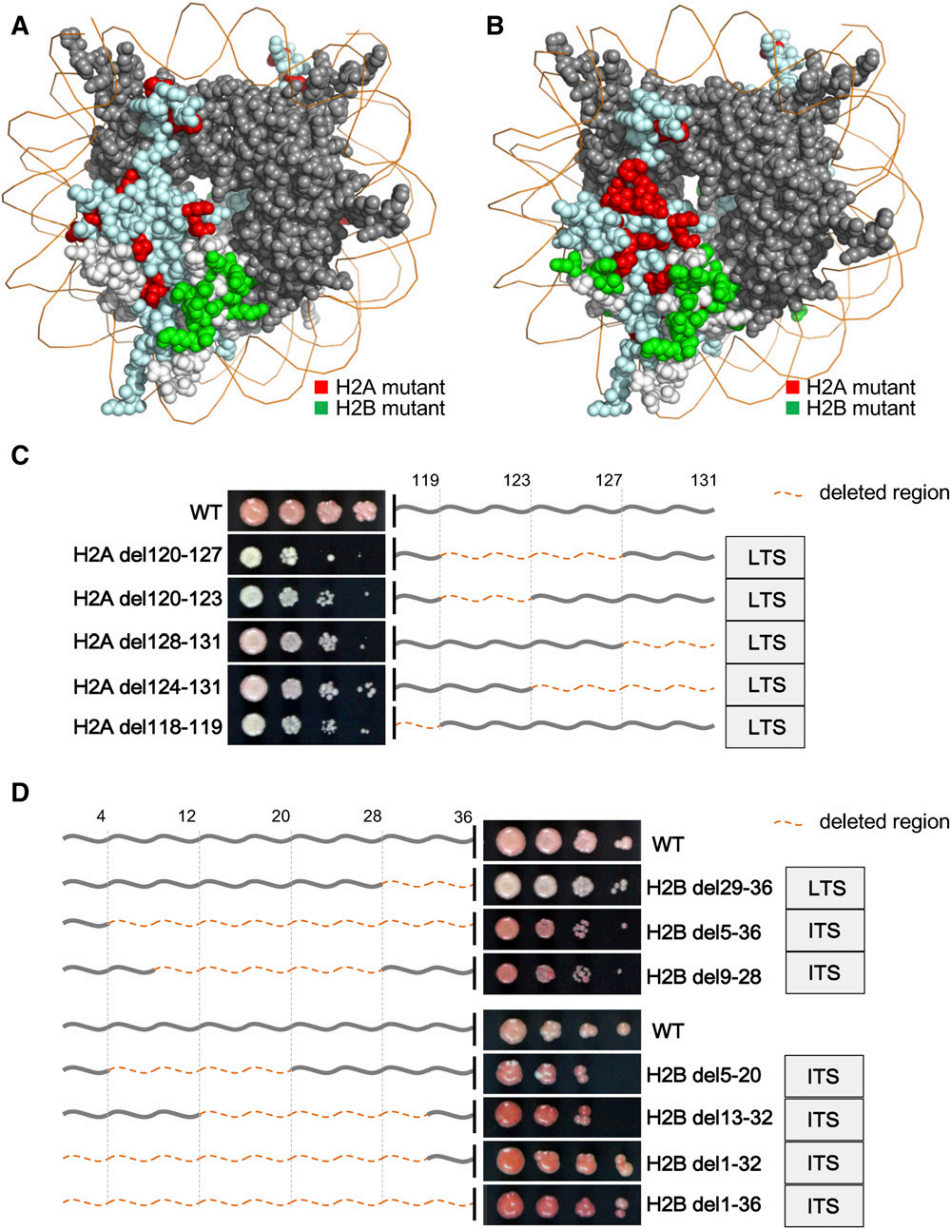
Identification of histone mutants affecting heterochromatin silencing. (A) Histone H2A and H2B mutants with LTS, except tail deletion mutants and the compound substitutions, are highlighted in the nucleosome structure. Bright red: H2A mutants. Bright green: H2B mutants. (B) Histone H2A and H2B mutants with LRS, except tail deletion mutants and the compound substitutions, are highlighted in the nucleosome structure with the same colors as in (A). (C) Telomere silencing defects of the histone H2A C-terminal tail deletion mutants. Dark gray lines represent the H2A C-terminal tail. The number of corresponding amino acids are marked above. The deleted regions are marked with orange dotted lines. (D) Telomere silencing phenotypes of the histone H2B N-terminal tail deletion mutants. Dark gray lines are used to represent H2B N-terminal tail. The number of corresponding amino acids are marked above. The deleted regions are marked with orange dotted lines as (C). ITS, increased telomere silencing; LRS, loss of rDNA silencing; LTS, loss of telomere silencing; WT, wild-type.

Mapping the identified residues onto nucleosome structure revealed the existence of shared and distinct regions between loss of telomere (LTS) and loss of rDNA silencing (LRS) phenotypes (Figure 4, A and B). Strikingly, most LTS mutants on histone H2B cluster together (Figure 4A), flanking a region known to directly interact with the BAH domain of Sir3 (Norris *et al.* 2008; Norris and Boeke 2010; Armache *et al.* 2011), suggesting that these mutants might compromise silencing by blocking the Sir3–nucleosome interaction. Furthermore, it is notable that nearly all these mutants also showed an LRS phenotype. Since Sir3 is not necessary for rDNA silencing, it is possible that other BAH-containing factors might mediate silencing within rDNA. Additionally, we found that five deletion mutants of the H2A C-terminal tail showed an obvious LTS phenotype (Figure 4C and Table S5 in File S1), including two that also showed an LRS phenotype (Table S5 in File S1). On the other hand, seven long deletion mutants of the H2B N-terminal (at least 16 amino acids deleted) displayed obviously increased telomere silencing (ITS), while another deletion mutant (H2B del29-36) showed an obvious LTS phenotype (Figure 4D and Table S5 in File S1). The dense thicket of silencing phenotypes mapping in a complex manner reveals not only the different behaviors of the two histone tails, but also the existence of distinct roles for silencing regulation within subregions of the H2B N-terminal tail.

### Profiling complex phenotypes by the bar-seq method

**Figure 5:**
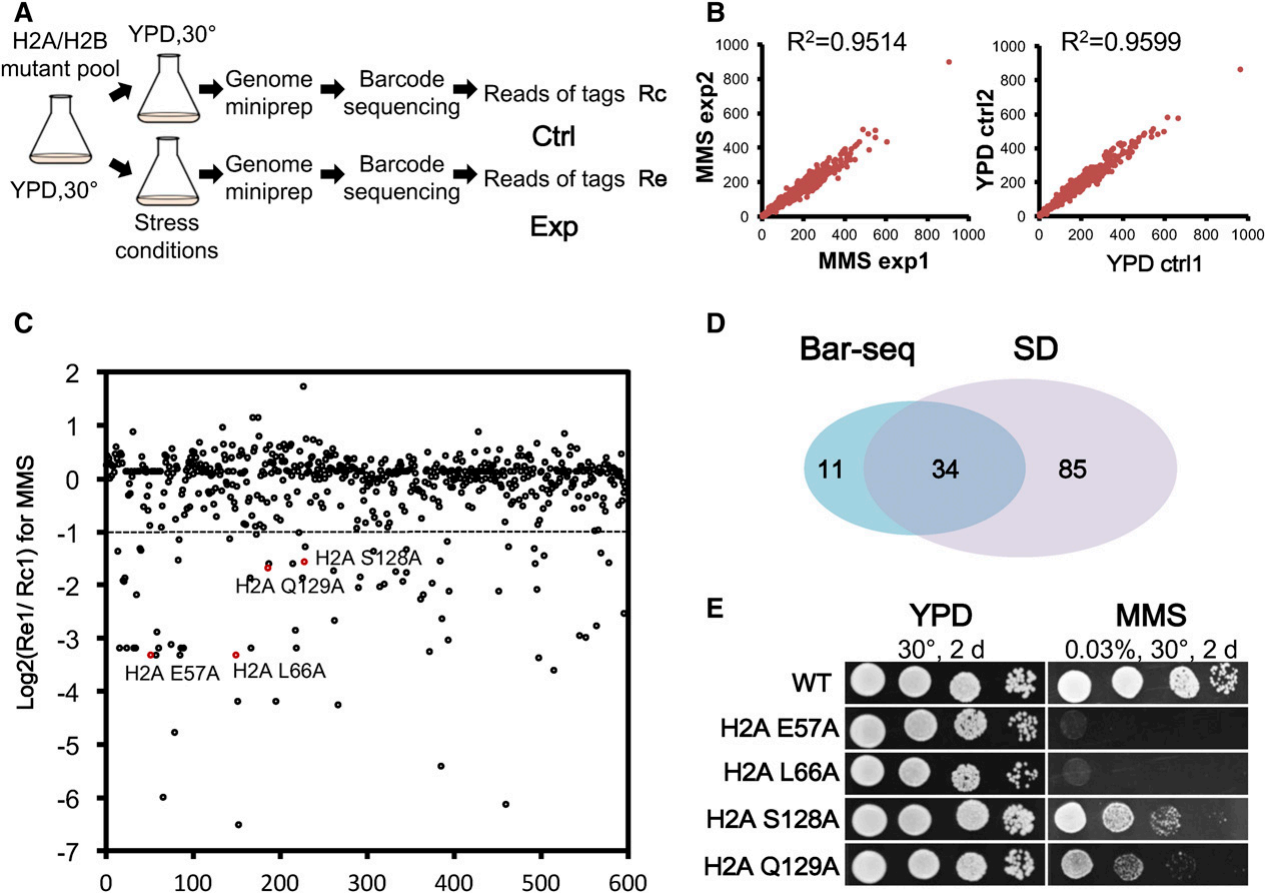
MMS sensitivity assay of histone mutants with barcode sequencing. (A) A schematic diagram to analyze stress sensitivities of pooled histone mutants by bar-seq. The mutants used in this assay were strains with a single copy of integrated histone genes (BY-H2ML1). (B) Correlation of the two replicates each for the Exp and Ctrl groups is indicated. (C) The sequence results for one of the MMS-treated mutant populations. The mutants with a log2 ratio below −1 were arbitrarily defined as MMS-sensitive candidates. (D) Comparison of identified MMS mutants identified by bar-seq and SD test for individual strains. The mutants used in both tests were strains with a single copy of integrated histone genes (BY-H2ML1). (E) Confirmation of MMS sensitivity of candidates identified by high-throughput sequencing using the SD test. bar-seq, barcode sequencing; Ctrl, control; Exp, experimental; MMS, methyl methanesulfonate; SD, serial dilution; WT, wild-type; YPD, yeast extract peptone dextrose.

The spotting assay commonly used to identify the sensitivity of a strain under certain stress conditions is simple and straightforward (Matsubara *et al.* 2007; Dai *et al.* 2008; Nakanishi *et al.* 2008; Sakamoto *et al.* 2009). However, with an increasing number of mutants, it takes a prolonged time and is also error-prone. With recent advances in sequencing technology, an efficient method for the analysis of pooled samples, highly-multiplexed bar-seq, or bar-seq, was developed (Smith *et al.* 2009, 2010), allowing the assessment of multiple samples in a single sequencing experiment. Given the presence of unique barcodes in the histone mutants, we adopted the multiplexed bar-seq method to analyze the composition of the histone mutants in up to 96 samples. As shown in Figure 5A, a pool of histone H2A and H2B mutant strains were cultured and divided into control and drug-treatment groups. After treatment, both pools were harvested, and the mutants within each pool were identified by high-throughput sequencing and compared. MMS was used as the drug for the demonstration. Figure 5B illustrates high concordance between two independent experiments in both MMS-treated (left panel) and untreated (right panel) samples. Figure 5C and Figure S5A in File S1 depict the sequencing reads from treated samples relative to the untreated controls. Mutants with a log2 ratio below −1 were defined as MMS-sensitive candidates. The two repeats predicted 56 or 50 candidates, respectively, and the 45 overlapping candidates (Figure S5B in File S1) were used for further analysis.

Next, we compared the bar-seq result with that from SD assays performed on each individual mutant. Figure 5D shows the Venn diagram between mutants identified by bar-seq and SD. The overall results from the two assays are largely in agreement; ∼75% of the mutants (34/45) were found in common despite the fact that distinct assay conditions were used (2 hr in liquid for bar-seq and several days on plates for the SD). To further confirm the bar-seq result, we randomly chose four H2A mutants and assayed them by a traditional SD test. The four mutants were: S128A and Q129A with a log2 ratio of about −1.5, which are defined as moderately sensitive mutants, and E57A and L66A with log2 ratio < −3.0, which are regarded as severely sensitive mutants. Figure 5E indicates that all four mutants are sensitive to MMS treatment and the degree of sensitivity correlates well with their log2 ratio in bar-seq. Therefore, bar-seq could be used to avoid phenotypic assays using individual mutants, which can be laborious.

## Discussion

The N-terminal tails of H3 and H4 are known hot spots of PTM and play many critical biological roles (Kouzarides 2007; Tan *et al.* 2011; Huang *et al.* 2014). Unlike histones H3 and H4, H2A and H2B each contain both a N-terminal tail and a C-terminal tail, which were all systematically assessed in this study. Similar to the results in Figure 2, the H2A N- and H2B C-termini did not show phenotypes in either the chromosome loss assay or silencing assay, whereas H2A C- and H2B N-terminal mutants showed phenotypes in both assays. Furthermore, although the mutants on both the H2A C- and H2B N-terminal tails showed defects in chromosome stability, silencing, and the response to DNA damage, they also showed distinct features, consistent with an underlying functional difference.

First, for the mutants with a chromosome loss phenotype, all three H2A C-terminal tail deletions were sensitive to microtubule drugs, while none of the five mutants on the H2B N-terminal tail showed such sensitivity (Figure 3A and Table S3), suggesting distinct mechanisms in maintaining genome integrity for the two tails. Although the H2A C-terminal tail is reportedly critical for docking Sgo1 and thereby maintaining regular chromosome segregation (Kawashima *et al.* 2010, 2011; Yamagishi *et al.* 2010), the mechanisms by which the H2B N-terminal tail maintains genome stability remain unclear. No point mutation in the H2B N-terminal tail showed an obvious chromosome loss phenotype, indicating a likely redundant function among these residues. From the results in Figure 3A, the region leading to a severe defect in genome stability was narrowed down to residues 29–36, which are in close proximity to the highly basic HBR domain (Parra *et al.* 2006). The same crucial region (sensitive to both DNA damage and temperature stresses) was mapped by the high-throughput screen (Figure 2 and Table S3). Taking the position of the HBR domain and mutant phenotypes together, we propose that the disruption of the interactions between basic residues in the HBR domain and DNA affected nucleosome functions by altering their structural stability. Consistent with this, deletion of the HBR domain causes increased nucleosome accessibility and decreased histone occupancy in the genome (Nag *et al.* 2010; Mao *et al.* 2016). In addition, the HBR domain has been reported to affect the activity of FACT in both nucleosome assembly and disassembly (Zheng *et al.* 2014; Mao *et al.* 2016). Since this domain is not essential for FACT binding (Mao *et al.* 2016), the attenuated interaction between HBR and DNA might provide a better explanation.

Second, mutants of the two tails showed dramatically different patterns of telomere silencing (Figure 4, C and D). Deletions of distinct regions on the H2A C-terminal tail led to silencing defects (Figure 4C and Table S5 in File S1), indicating important roles of the residues on the H2A C-terminus in the repression of silent chromatin. At the same time, S121D and S121A also showed LTS and LRS phenotypes, respectively. Although phosphorylation of serine 121 is critical for chromosome segregation (Kawashima *et al.* 2010; Yamagishi *et al.* 2010), how this residue is involved in silencing remains unclear. For H2B N-terminal tail mutants, del29-36 showed both LTS and LRS phenotypes, consistent with previous results of HBR-deleted strains (Parra *et al.* 2006). Such derepression might result from decreased histone occupancy in chromatin (Mao *et al.* 2016). Additionally, seven H2B N-terminal tail deletion mutants showed a paradoxical ITS (Figure 4D and Table S5 in File S1). Among them, four mutants had the truncated H2B N-terminal tail in which part or all of the HBR domain was also deleted, such as del1-32 and del5-36. The opposite effects on silencing for overlapping deletions strongly suggests the existence of antagonistic mechanisms within distinct subregions of the H2B N-terminal tail. Based on the literature, six lysine residues (K6, K11, K16, K17, K21, and K22) are acetylated and maintain hypoacetylation in silencing mating loci and telomeric regions (Roth *et al.* 2001; Suka *et al.* 2001). It is possible that some combination of modifications and the resulting changes in charge on the varying numbers of lysine residues underlie these opposing silencing phenotypes. However, H2B K6,11,16Q, and H2B K22E, also showed ITS phenotypes (Table S5 in File S1), complicating mechanistic interpretation.

Third, from the results of DNA damage stresses, the residues from 128 to 131 on H2A were sensitive to MMS but resistant to HU, while no regions in H2B showed this type of pattern (Table S3 and Table S4 in File S1). In the nearby C-terminal motif, there is the earliest mark of the double strand break: γH2AX (Downs *et al.* 2000; Keogh *et al.* 2006). Mutants in this motif result in defects in downstream pathways. Although MMS and HU can both lead to DNA damage, the cellular responses to the two drugs are distinct. It has been reported the dephosphorylation of Rad53 by Pph3 is required for checkpoint inactivation after MMS but not HU exposure (Wang *et al.* 2012), while Glc7 is needed for the disappearance of hyperphosphorylated Rad53 to recover from HU but not MMS exposure (Bazzi *et al.* 2010). In addition, some other evidence supports the idea that different phosphorylation states of Rad53 may exist under different toxic stresses and that these patterns are regulated by the activities of various kinases and phosphatases (Smolka *et al.* 2005; Keogh *et al.* 2006; Yao *et al.* 2017). Further studies to unravel how exactly these enzymes are engaged in response to different regents will be required.

These versatile next-generation histone mutant libraries promise to uncover many additional phenotypes associated with DNA packaging, transcription, replication, and repair, and are being made publicly available.

## Supplementary Material

Supplemental material is available online at www.g3journal.org/lookup/suppl/doi:10.1534/g3.117.300252/-/DC1.
