# Supplementary material for: Dissecting Nucleosome Function with a Comprehensive Histone H2A and H2B Mutant Library
Source: G3 (Bethesda). 2017 Oct 16;7(12):3857–66. doi: 10.1534/g3.117.300252 (PMC5714483; doi:10.1534/g3.117.300252)
Supplement: Supplementary file 2 [file 3857FileS1.docx]

Supplementary data:

**Dissecting nucleosome function with a comprehensive histone H2A and H2B mutant library**

Shuangying Jiang^1,2,#^, Yan Liu^1,#^, Caiyue Xu^1,$^, Yun Wang^3,4^, Jianhui Gong^3,4^, Yue Shen^3,4^, Qingyu Wu^1^, Jef D. Boeke^5^ and Junbiao Dai^1,2*^

^1^ MOE Key laboratory of Bioinformatics and Center for Synthetic and Systems Biology, School of Life Sciences, Tsinghua University, Beijing 100084, PR China

^2^ Center for Synthetic Biology Engineering Research, Shenzhen Institutes of Advanced Technology, Chinese Academy of Sciences, Shenzhen 518055, China

^3^ China National GeneBank, BGI-Shenzhen, Shenzhen 518120, China

^4^ BGI-Shenzhen, Shenzhen, 518083, China

^5^ Institute for Systems Genetics and Department of Biochemistry and Molecular Pharmacology, New York University Langone Medical Center, New York, New York 10011, USA

**Running title**: Histone H2A and H2B mutagenesis

**Keywords**: histone; heterochromatin gene silencing; DNA damage, Post-translational Modification

^#^ These authors contributed to this work equally

$ Current address: Epigenetics Program, Department of Cell and Developmental Biology, Perelman School of Medicine, University of Pennsylvania, Philadelphia, Pennsylvania 19104, USA

**Corresponding author:**

Dr. Junbiao Dai

Center for Synthetic Biology Engineering Research, Shenzhen Institutes of Advanced Technology, Chinese Academy of Sciences, Shenzhen 518055, China.

Phone: 86-755-86585244; Email: junbiao.dai@siat.ac.cn


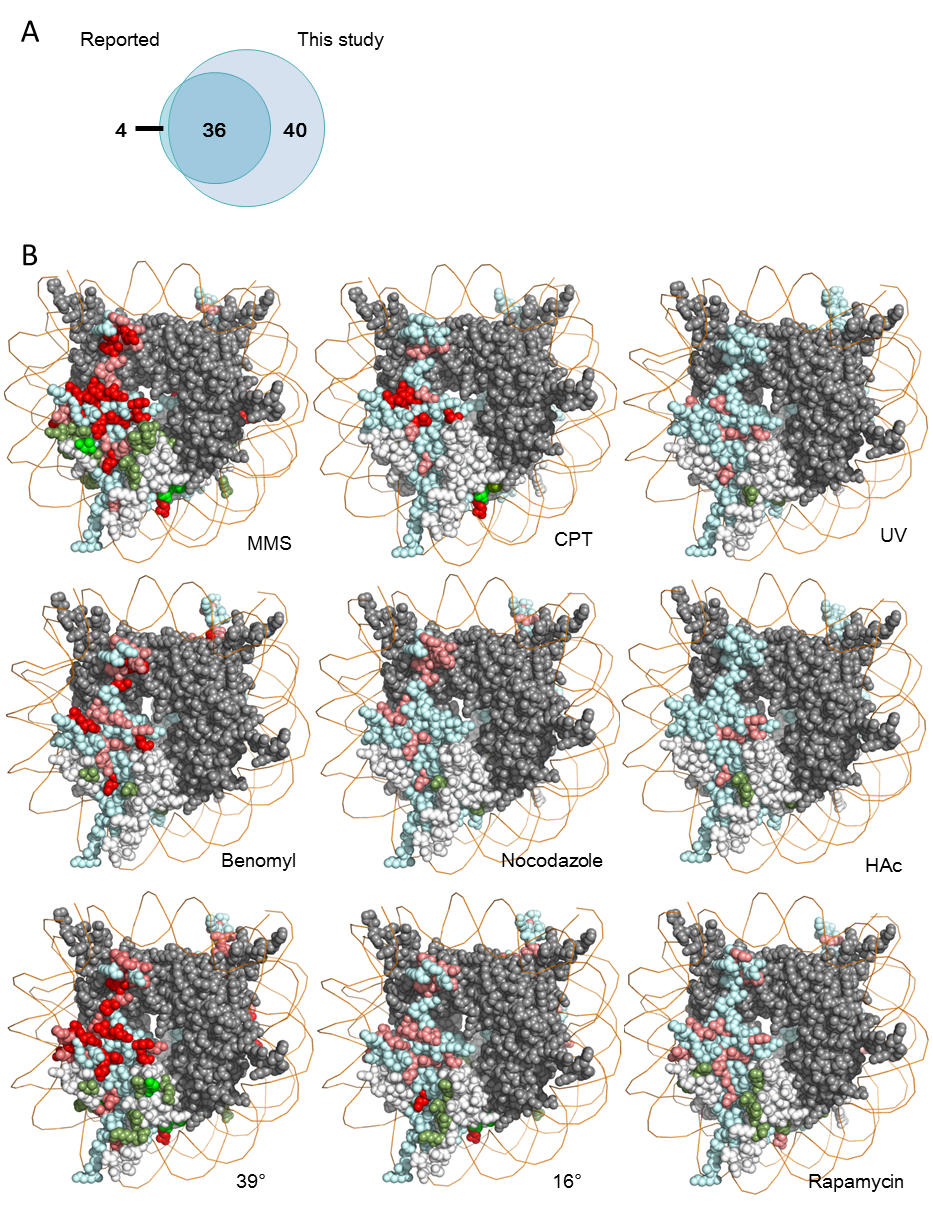


**Figure S1. A functional map of H2A/H2B.**

**A.**Comparison of important residues for MMS sensitivity identified before ([Matsubara *et al.* 2007](#_ENREF_23); [Sakamoto *et al.* 2009](#_ENREF_40)) and in this study.

**B.** Mutants sensitive to different stresses were marked with different colors (see Figure 1B for explanation), excepting the mutants on histone tails and the compound substitutions. If two or more mutants for a certain residue showed phenotypes, the highest phenotype degree of this residue was displayed.


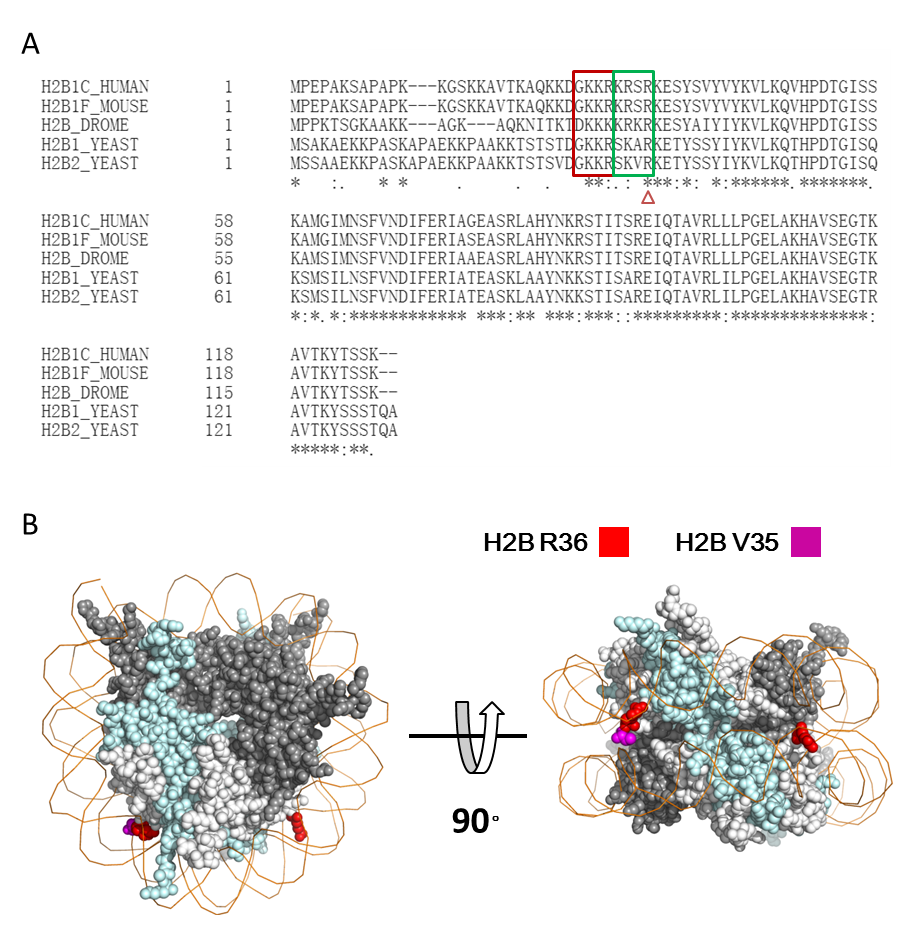


**Figure S2. The XKXR motif is very important and reveals many interesting phenotypes, as described in the main text.**

**A.** The alignment of H2B from yeast to human. The alignment was done with Clustal W. Amino acids from 29 to 32 are marked with a red square and the ones from 33 to 36 are marked with a green square. R36 is indicated by a red triangle.

**B.** The location of H2B V35/R36 in the nucleosome structure.

**
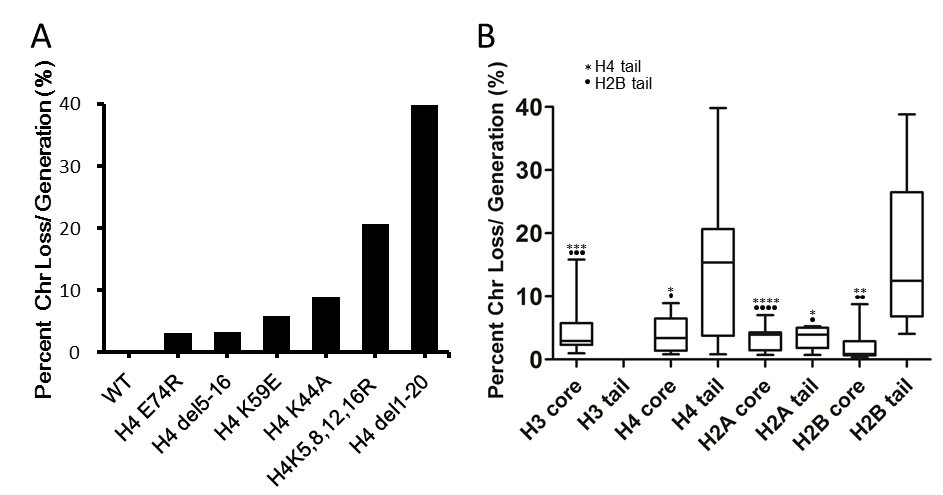
**

**Figure S3. Mutants of H4 tail and H2B tail showed more severe phenotypes than other mutants.**

**A.** Several additional H4 mutants with chromosome loss phenotype, which were not revealed in previous screens were identified.

**B.** Mutants on H4 tail and H2B tail showed higher chromosome loss rates than other mutants. The chromosome loss rates of different groups were showed with box (min-max) and compared with H4 tail (*) and H2B tail (●) using unpaired t test.. * or ●means P<0.05, ** or ●● means P<0.01. *** or ●●● means P<0.001. **** or ●●●● means P<0.0001. There is no significant difference between H4 tail and H2B tail.


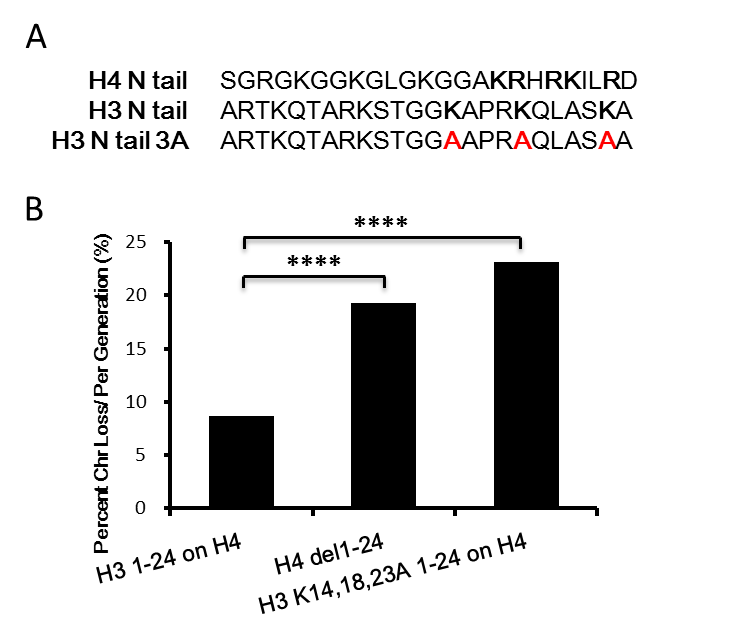


**Figure S4. Positively charge amino acids on the H4 N tail are especially important for genome stability.**

**A** The sequences for the first 24 amino acids of different histone tails. The lysine residues mutated in panel B are marked in red.

**B** The frequency of chromosome loss per generation for different H4 mutants. ****means P<0.0001.


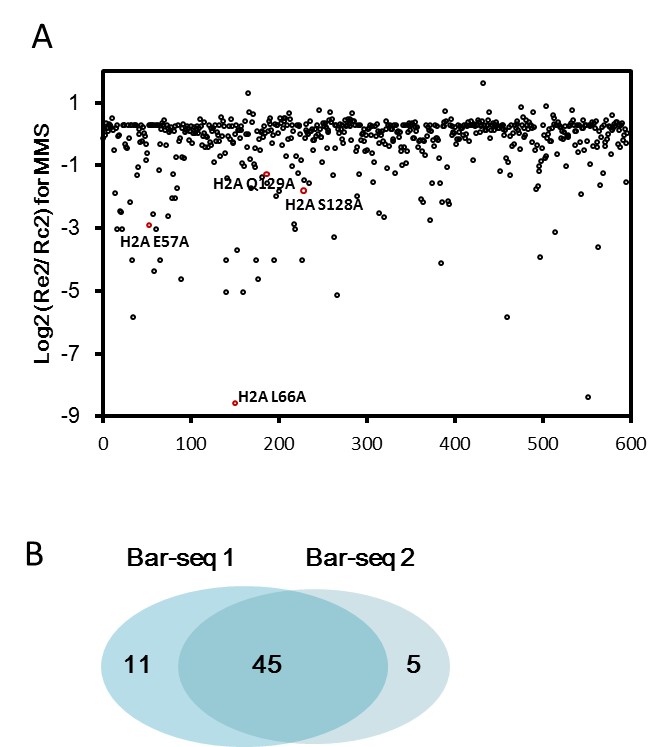


**Figure S5. MMS sensitive candidates from two repeats of bar-seq analysis largely overlap.**

**A** The sequence results of a second MMS-treated mutant population. The mutants with log2 ratio below -1 were defined as MMS-sensitive candidates.

**B** Comparison of the candidates from two repeats. Bar-seq 1: the candidates from Figure 5C. Bar-seq 2: the candidates from panel A.

**Table S1. Strains used in this study**

| **Strains** | **Genotype** |
| --- | --- |
| BY-H2ML1&2 | *MATα, his3∆200, leu2∆0, lys2∆0, trp1∆63, ura3∆0, met15∆0, hta2-htb2::NatMX4-HTAS-HTBS, hta1-htb1::LEU2-HTAS-HTBS* |
| SHY15 | *MATα,* *ura3∆0, Leu2∆0 , his3-112, trp1-1, can1-100, ade2-1, bar1-1, SUP11 CFIII(CEN3, YPH983):HIS3, hta2-htb2::HygMX4* |
| JDY187 | *MATα,* *his3∆200, leu2∆1, trp1∆63,lys2∆0, ura3-167, met15∆0, ade2::his, RDN1::Ty1-MET15, TELV::ADE2, hta2-htb2::HygMX4, hta1-htb1::NatMX4, CEN-URA3-HTA2-HTB2(pJD78)* |
| BY-H2ML1 | *MATα, his3∆200, leu2∆0, lys2∆0, trp1∆63, ura3∆0, met15∆0, hta2-htb2::HygMX4, hta1-htb1::LEU2-HTAS-HTBS* |
| JDY176 | *MATa, ura3Δ0, Leu2-3,11, his3-11, trp1-1, can1-100, ade2-1, bar1-1, SUP11*  *CFIII (CEN3, YPH983):HIS3, hht1-hhf1::NatMX4* |
| H2ML1 | H2A/H2B mutant library (plasmid) which can be integrated into *HTA1-HTB1* loci |
| H2ML2 | H2A/H2B mutant library (plasmid) which can be integrated into *HTA2-HTB2* loci |
| H3/4ML2 | H2A/H2B mutant library (plasmid) which can be integrated into *HHT2-HHF2* loci |
| pJD230 | Chimeric mutation of H4 (plasmid): H3 1-24 on H4 |
| pJS14 | Chimeric mutation of H4 (plasmid): H3 K14,18,23A 1-24 on H4 |

**Table S2.** **The different assays for high-throughput phenotyping of yeast library with double-copy histones.**

| **Class** | **Assay** |
| --- | --- |
| DNA damage stresses | HU |
|  | CPT |
|  | UV |
|  | MMS |
| Drugs that disrupt microtubules | Benomyl |
|  | Nocodazole |
| Temperature stresses | 39° |
|  | 16° |
| Other stresses | Rapamycin |
|  | Acetic acid |

**Table S3. The phenotype database for yeast library with double-copy H2A/H2B mutants.**

(Provided as an Excel spreadsheet)

**Table S4. Mutants only sensitive to MMS stress**

| H2A mutants | K4R,K7R,K13R |
| --- | --- |
|  | R36E |
|  | R43A |
|  | R89E |
|  | N90A |
|  | P110A |
|  | **S128A** |
|  | **S128D** |
|  | **Q129A** |
|  | **Q129E** |
|  | **L131A** |
|  | **DEL128-131** |
| H2B mutants | DEL17-32 |
|  | DEL25-36 |
|  | I44A |
|  | T55D |
|  | F73A |
|  | E79A |
|  | Y86F |
|  | K89Q |
|  | E108A |
|  | E108Q |
|  | K111E |

Bold face indicates the mutants concentrated in the H2A C tail.

**Table S5. Mutants with abnormal silencing in telomeres and rDNA**

|  | **Loss of telomere silencing (LTS)** | **Increase of telomere silencing(ITS)** | **Loss of rDNA silencing (LRS)** |
| --- | --- | --- | --- |
| H2A | F26A, R30A, R33E, I44A, **E57A, E65A, E65Q**, K76A, R78A, R82E, **D92R, E93A, E93Q**, H113A, *L116A, P118V, S121D*  *del118-119, del120-127, del120-123, del124-131, del128-131* | S15D, A61S, L64A, N69D, R72A, R72E, N74A, I79A, I80A, R82A, R82K, I88A, N90A | S19D, R33E, P49V, V50A, Y51A, Y58F, L64A, **E65A, E65Q**, L66A, A70S, R82E, Q85E, L86A, R89E, N90D, **D92N, E93A, E93Q**, L94A, I103A, G107A, L109A, P110V, H113Q, *S121A, del120-123, del120-127* |
| H2B | S67D, L83A, Y86E, **R95A, R95E, R95K, Q98E, T99D, R102E, K111A, K111E, K111Q, S115D, E116A, T118D, R119A, R119E, R119K**, T122D, K123A, K123E, K123Q, K123R, del29-36 | K22E, T51D, H52A, P53A, E96Q, E96R, T99A, L103A, E108A, E108Q, S115A, K6Q, K11Q, K16Q,  del1-20, del1-32, del13-32, del1-36, del5-20, del5-36, del9-28 | K11A, R36E, T51D, H52Q, D54R, S63A, I64A, L65A, S67D, D71N, L83A, K89E, **Q98A, Q98E, T99A, T99D, R102A, R102E, R102K,** G107A, **K111A, K111E, K111R, H112A, S115D,** G117A, **T118A, T118D, R119A, R119E, R119K**, T122A, T122D, K123A, K123E, K123Q, K123R, K6Q, K11Q, K16Q, del21-36, del29-36 |

Bold letter: residues flanking the region known to directly interact with the BAH domain of Sir3

Italic letters: residues near H2A C terminal

**Table S6. Primers for barcode sequencing.**

| 1^st^ round PCR for TAG sequencing | F1 | CACGACGCTCTTCCGATCT*NNNNN*atgtccacgaggtctct |
| --- | --- | --- |
|  | **R1** | AGCAGAAGACGGCATACGAGCTCTTCCGATCTcggtgtcggtctcgtag |
| **2^nd^ round PCR for TAG sequencing** | **F2** | AATGATACGGCGACCACCGAGATCTACACTCTTTCCCTACACGACGCTCTTCCGATCT |
|  | **R2** | CAAGCAGAAGACGGCATACGA |

*NNNNN*: The index sequence for different samples.

nnnnn: The universal annealing sequences to amplify TAGs.

NNNNN: The sequencing adaptors P5/ P7.
